# Supplementary material for: Physical and environmental drivers of Paleozoic tetrapod dispersal across Pangaea
Source: Nat Commun. 2018 Dec 6;9:5216. doi: 10.1038/s41467-018-07623-x (PMC6284015; doi:10.1038/s41467-018-07623-x)
Supplement: Supplementary file 1 — Description of Additional Supplementary Files [file 41467_2018_7623_MOESM1_ESM.pdf]

Description of Additional Supplementary Files

File Name: Supplementary Data 1
Description: The source trees used to generate the supertree, and the clade for which they provide relationships

ile Name: Supplementary Data 2
Description: Total supertree in phylip format

File Name: Supplementary Data 3
Description: Age ranges of the taxa, giving the maximum and minimum estimates for the first and last appearances

File Name: Supplementary Data 4
Description: Presence/Absence matrix giving the geographic range of every taxon. Note that postPaleozoic lineages with ghost lineages extending back into the Paleozoic were collapsed into larger clades and were not included in the biogeographic analysis, as their ancestral areas are as yet unknown. 1=present in the area, 0=absent from that area WEU = Western Europe; WNA = Western North America; ENA= Eastern North America; EEU = Eastern Europe; EAS = Eastern Asia; NSA = Northern South America; NAF = Northern Africa; SSA = Southern South America; SAF = Southern Africa; ANT = Antarctica; MAD = Madagascar; IND = India; AUS = Australia

File Name: Supplementary Data 5
Description: Code used to simulate a biogeographic history over a phylogeny in R. Required parameters and their explanations provided in the code's annotations.
