# Supplementary material for: Physical and environmental drivers of Paleozoic tetrapod dispersal across Pangaea
Source: Nat Commun. 2018 Dec 6;9:5216. doi: 10.1038/s41467-018-07623-x (PMC6284015; doi:10.1038/s41467-018-07623-x)
Supplement: Supplementary file 3 — Supplementary Information [file 41467_2018_7623_MOESM3_ESM.pdf]

Supplementary Information

Physical and environmental drivers of Paleozoic tetrapod dispersal across Pangaea

Brocklehurst et al.

Supplementary Note 1

Supertree generation

The most recent and comprehensive phylogenies of each major clade were selected as source tree, and several "backbone" trees were used to establish the relationships of these clades relative to each other. A few of smaller, less recent trees were included as the only analyses to include specific taxa. To be eligible for inclusion, a phylogenetic analysis should: A) include at least three taxa from the time period under study (Carboniferous and Permian); B) have been generated using a cladistic/Bayesian/likelihood computer algorithm (composite trees, manually generated phylogenies, supertrees and supermatrices were not included); C) be present in a publication giving full details of the analytical methods. The full list of source trees is given in Supplementary Data 1

Supplementary Note 2

Defemination of bioregions

The taxa in the supertree were assigned to one of 13 bioregions. These regions, and the boundaries defining them, are detailed here.

1. Western North America. This represents the North American localities west of the internal Hueco seaway [1]. This includes localities from New Mexico, Utah, Arizona and Colorado.
2. Eastern North America. This represents the North American localities east of the Hueco seaway. While at that time geographically continuous with western Europe and northern South America, this bioregion is separated from the former by the Variscan orogeny and the latter by the Appalachides [2-4].

3. Northern South America. This represents the localities of the Parnaíba basin. Its separation from the southern South American bioregion is determined by palaeolatitude (25ºS), separating the palaeoequatorial and palaeotemperate localities. While at the time geographically continuous with northern Africa, the bioregions are separated by the southern extension of the Mauritenides [4,5].
4. Southern South America. This represents the palaeotemperate localities of the Paraná basin.
5. Western Europe. Includes localities mostly from France and Germany, also from the UK and Poland. These primarily represent basins within the Variscan orogeny [6]. Due to the orientation of Eurasia at that time, the separation between western and eastern Europe is latitudinal (20ºN), separating the palaeoequatorial localities of western Europe from palaeotemperate eastern European localities.
6. Eastern Europe: Includes localities from European Russia, Kazakstan and Tajikistan. Is geographically continuous with the east Asian localities, but separated from this bioregion by the Uralides [2].
7. Eastern Asia: mostly Chinese localities from the Ordos basin.
8. Northern Africa: Localities from Niger and Morocco. At that time was geographically continuous with western Europe but separated from these localities by the Variscan Orogeny [2-4]. Separation from Southern Africa is latitudinal (25ºS), separating the palaeoequatorial and palaeotemperate localities.
9. Southern Africa: Palaeotemperate basins, mostly including specimens from the Karoo of South Africa, but also the similar contemporary faunas from Tanzania, Zambia and Malawi.
10. Antarctica: Localities from the Fremouw Formation. Triassic in age, but several taxa have ghost lineages extending into the Permian so was included. Separated

from other bioregions both latitudinally (being within the Antarctic circle) and physically (representing a foreland basin within the Gondwanides) [7].

11. Madagascar: The Sakamena Formation is a marine basin representing a shallow internal seaway that is used in this analysis to separate the southern African and Indian bioregions
12. India: This includes the localities of the Pranhita-Godovari basin (while other Permian tetrapod-bearing localities are known from India, none of their taxa have been included in phylogenetic analyses and so they are not analysed here).
13. Australia. A single tetrapod-bearing formation is known from the Carboniferous-Permian of Australia: the Duckabrook Formation.

Supplementary Table 1

Mean likelihood, free parameter estimates, and Akaike Information Criterion values obtained across the 100 trees for the DEC, DIVA and BayeArea biogeographic models

	Log Likelihood	d	E	AIC
DEC	-1884.4529	0.01057	0.02177	3772.906
DIVA	-1863.7979	0.01127	0.01969	3731.596
BayeArea	-2064.1831	0.01267	0.05510	4132.366

Supplementary Table 2

Area adjacency matrix used in the BioGeoBEARS analysis. 1 indicates areas are adjacent. WEU = Western Europe; WNA = Western North America; ENA= Eastern North America; EEU = Eastern Europe; EAS = Eastern Asia; NSA = Northern South America; NAF = Northern Africa; SSA = Southern South America; SAF = Southern Africa; ANT = Antarctica; MAD = Madagascar; IND = India; AUS = Australia

	EUR	WNA	ENA	RUS	CHI	NSA	NAF	SSA	SAF	ANT	MAD	IND	AUS
**EUR**	1	0	1	1	0	0	1	0	0	0	0	0	0
**WNA**	0	1	1	0	0	1	0	0	0	0	0	0	0
**ENA**	1	1	1	0	0	1	1	0	0	0	0	0	0
**RUS**	1	0	0	1	1	0	0	0	0	0	0	0	0
**CHI**	0	0	0	1	1	0	0	0	0	0	0	1	1
**NSA**	0	1	1	0	0	1	1	1	0	0	0	0	0
**NAF**	1	0	1	0	0	1	1	0	1	0	0	0	0
**SSA**	0	0	0	0	0	1	0	1	1	0	0	0	0
**SAF**	0	0	0	0	0	0	1	1	1	1	1	1	0
**ANT**	0	0	0	0	0	0	0	0	1	1	0	1	1
**MAD**	0	0	0	0	0	0	0	0	1	0	1	1	0
**IND**	0	0	0	0	1	0	0	0	1	1	1	1	1
**AUS**	0	0	0	0	1	0	0	0	0	1	0	1	1

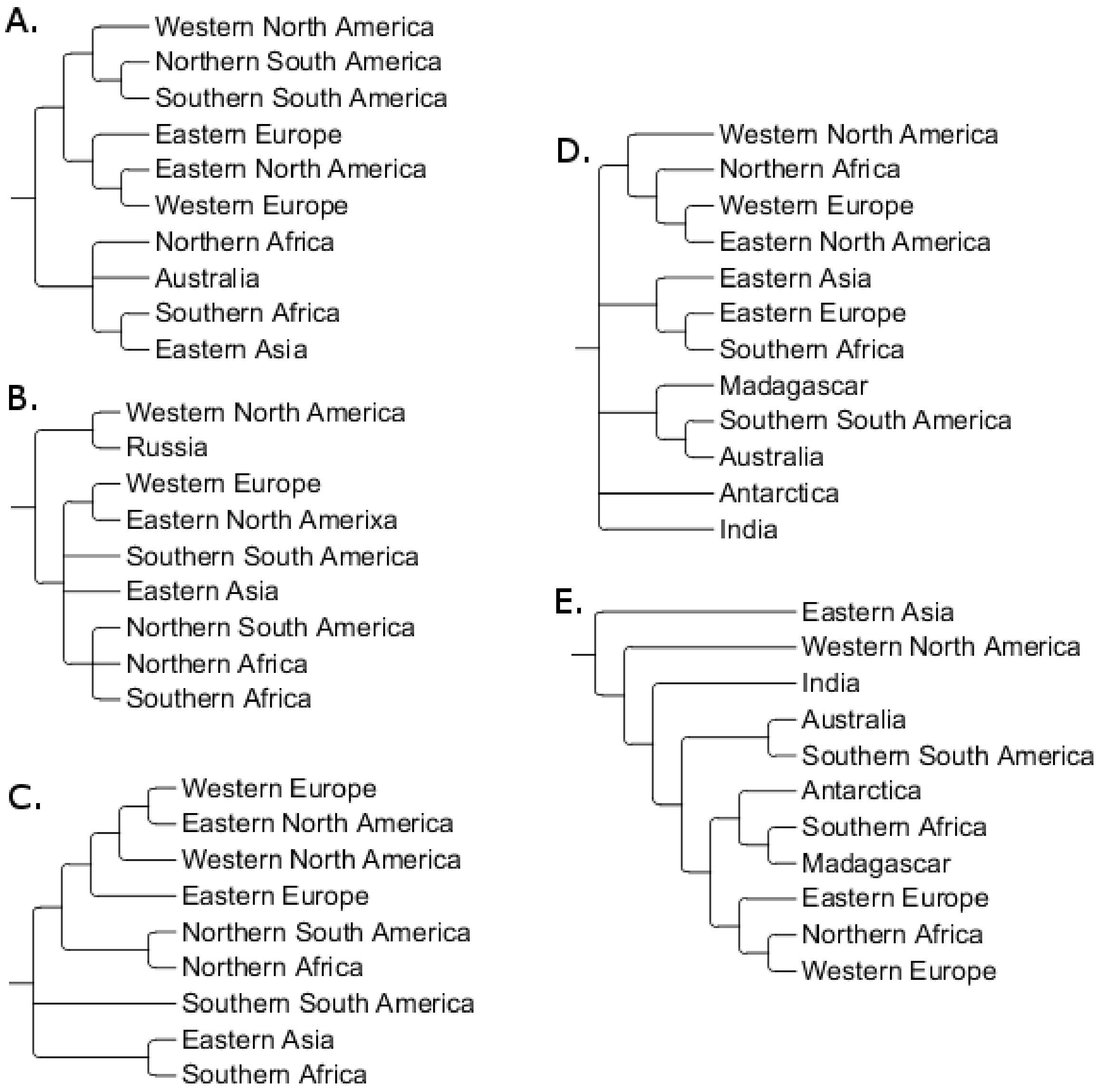

Supplementary Figure 1

Area cladograms obtained from the treefitting analysis from each time sliced supertree. A) Mississippian; B) Pennsylvanian; C) Cisuralian; D) Guadalupian; E) Lopingian

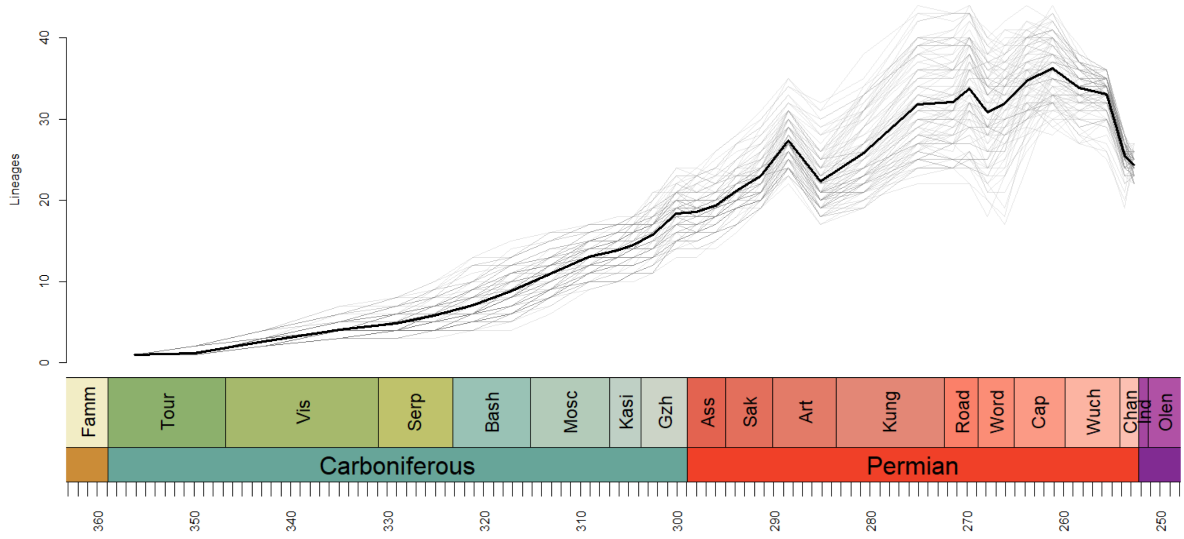

Supplementary Figure 2

Linage through time plot of Parareptilia. The thin translucent grey lines represent the number of lineages in each time bin calculated from each of the 100 time calibrated trees. The thick black line represents the mean diversity estimate.

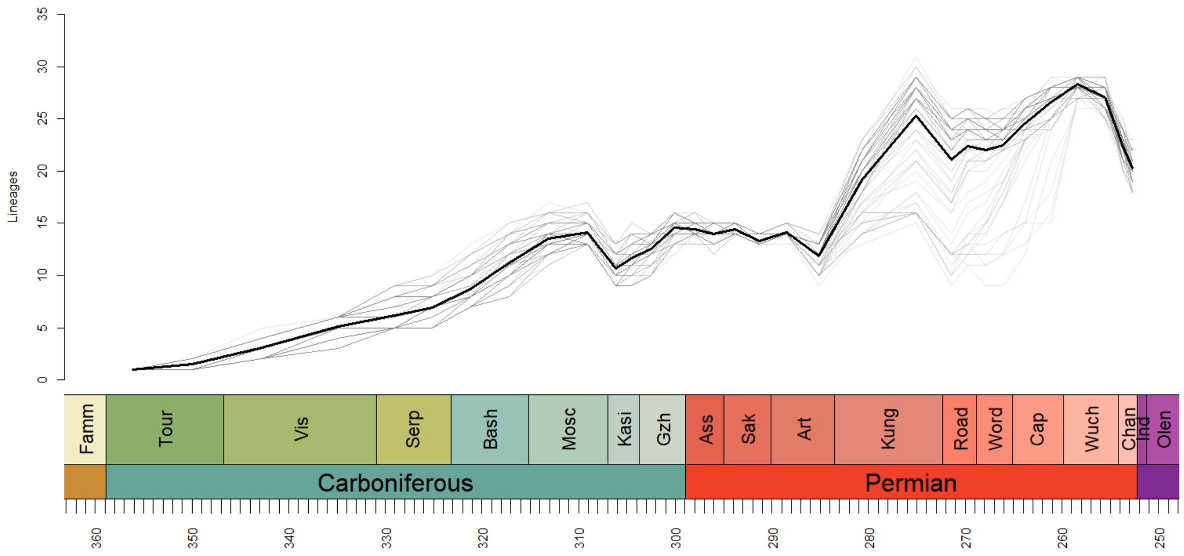

Supplementary Figure 3

Linage through time plot of Eureptilia. The thin grey lines represent the number of lineages in each time bin calculated from each of the 100 time calibrated trees. The thick black line represents the mean diversity estimate.

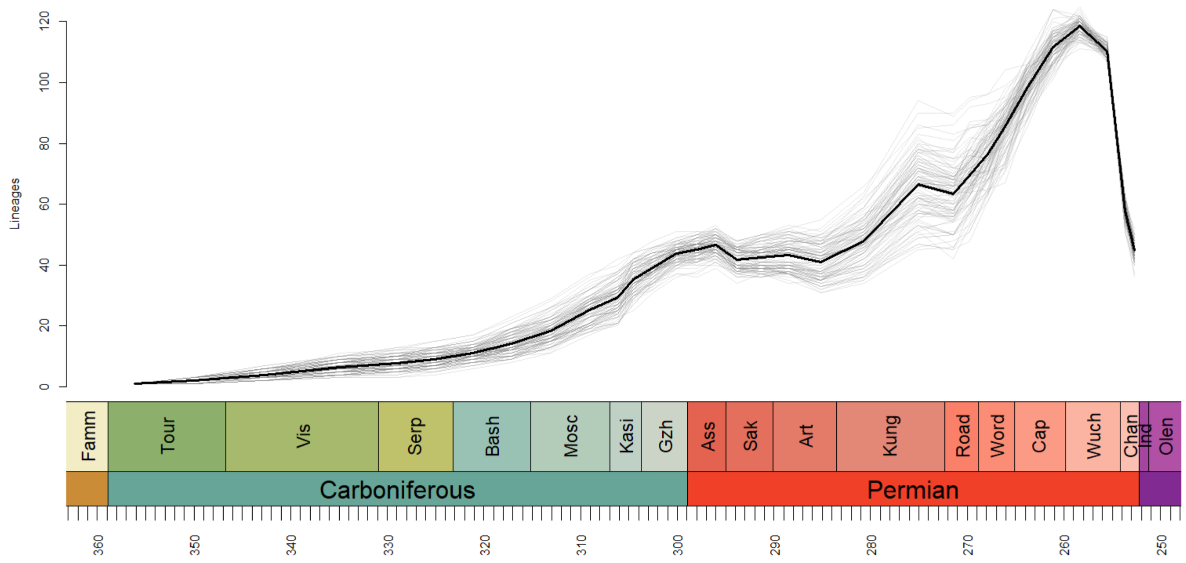

Supplementary Figure 4

Linage through time plot of Synapsida. The thin grey lines represent the number of lineages in each time bin calculated from each of the 100 time calibrated trees. The thick black line represents the mean diversity estimate.

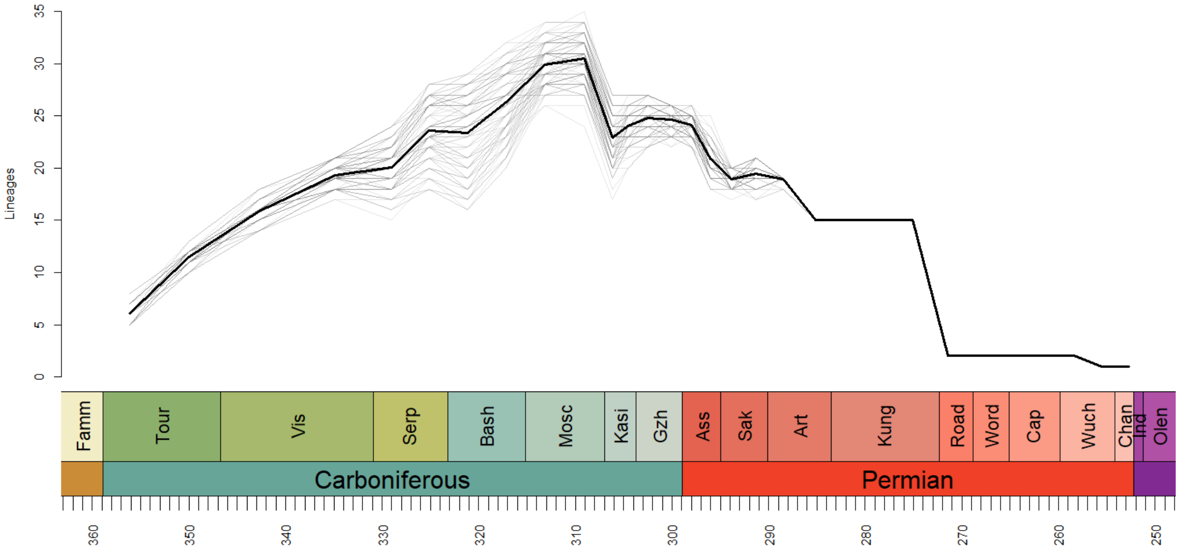

Supplementary Figure 5

Linage through time plot of Lepospondyli. The thin grey lines represent the number of lineages in each time bin calculated from each of the 100 time calibrated trees. The thick black line represents the mean diversity estimate.

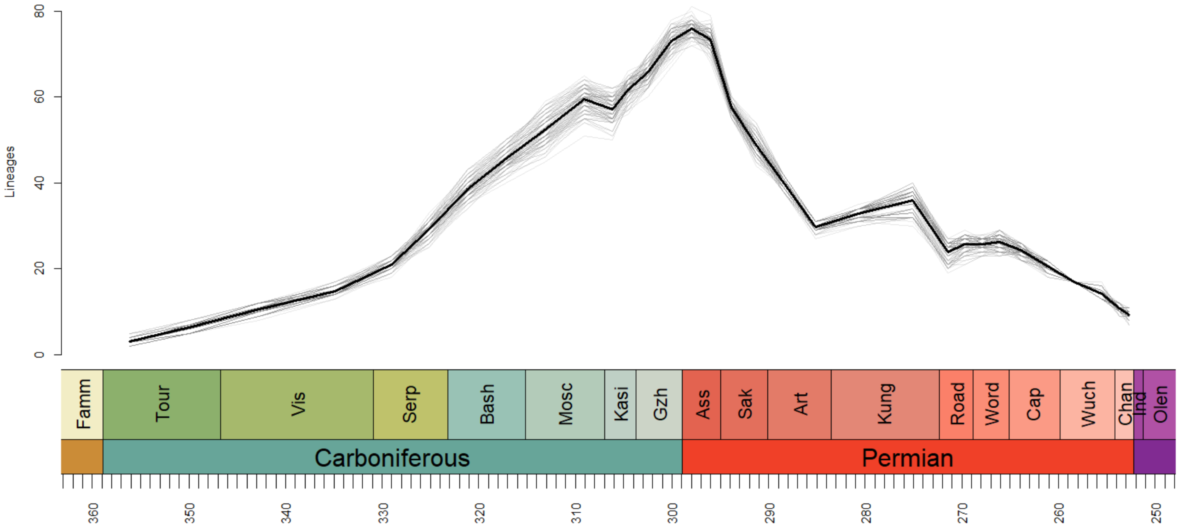

Supplementary Figure 6

Linage through time plot of Temnospondyli. The thin grey lines represent the number of lineages in each time bin calculated from each of the 100 time calibrated trees. The thick black line represents the mean diversity estimate.

Supplementary References

1. Lucas, S.G. *Traces of a Permian Seacoast: Prehistoric Trackways National Monument*. New Mexico Museum of Natural History and Science. (2011)
2. Rowley, D.B. et al. Carboniferous paleogeographic, phytogeographic, and paleoclimatic reconstructions. *Int. J. Coal Geol.* **5**, 7-42 (1985).
3. Arthaud, F., Matte, P... Late Paleozoic strike-slip faulting in southern Europe and northern Africa: Result of a right-lateral shear zone between the Appalachians and the Urals. *Geol. Soc. Am. Bul* **88**, 1305–1320. (1977)
4. Cisneros, J.C., Abdala, F., Atayman-Güven, S., Rubidge, B.S., Şengör, A.C.,Schultz, C.L.. Carnivorous dinocephalian from the Middle Permian of Brazil and tetrapod dispersal in Pangaea. *Proc. Nat Acad. Sic.* **109**, 1584-1588 (2012)
5. Şengör, A.C., Atayman, S. The Permian extinction and the Tethys: an exercise in global geology. *Geol. Soc Am. Spec Paper* **448**, 1-97 (2009)
6. Cleal, C.J. et al. Late Moscovian terrestrial biotas and palaeoenvironments of Variscan Euramerica. *Neth. J. Geosci.* **88**, 181-278 (2009).
7. Collinson, J.W., Isbell, J.L., Elliot, D.H., Miller, M.F., and Miller, J.G.M. Permian-Triassic Transantacrtic Basic. Pp 173-222 in J. J. Veevers and C.M. Powell (eds) *Permian-Triassic Pangaean basins and Foldbelts along the Panthalassan Margin of Gondwanalana.* Geological Society of America Memoir 184 (1994)
